# Design and methods of the prevalence and pharmacogenomics of tenofovir nephrotoxicity in HIV-positive adults in south-western Nigeria study

**DOI:** 10.1186/s12882-020-02082-3

**Published:** 2020-10-16

**Authors:** Muzamil O. Hassan, Raquel Duarte, Victor O. Mabayoje, Caroline Dickens, Akeem O. Lasisi, Saraladevi Naicker

**Affiliations:** 1 Department of Internal Medicine, Ladoke Akintola University of Technology Teaching Hospital, Osogbo, Nigeria; 2 Department of Medicine, Obafemi Awolowo University, Ile-Ife, Osun State Nigeria; 3 Internal Medicine Research Laboratory, Department of Internal Medicine, Faculty of Health Sciences, University of the Witwatersrand, Johannesburg, South Africa; 4 Department of Haematology, Ladoke Akintola University of Technology Teaching Hospital, Osogbo, Nigeria; 5 Department of OtoRhinoLaryngology, University College Hospital, Ibadan, Nigeria; 6 Department of Internal Medicine, Faculty of Health Sciences, University of the Witwatersrand, Johannesburg, South Africa

**Keywords:** Tenofovir, Kidney tubulopathy, HIV, Pharmacogenomics, *APOL1*, Drug transporters

## Abstract

**Background:**

Individuals of African descent are at higher risk of developing kidney disease than their European counterparts, and HIV infection is associated with increased risk of nephropathy. Despite a safe renal profile in the clinical trials, long-term use of tenofovir disoproxil fumarate (TDF) has been associated with proximal renal tubulopathy although the underlying mechanisms remain undetermined. We aim to establish the prevalence of and risk factors for TDF-induced kidney tubular dysfunction (KTD) among HIV-I and II individuals treated with TDF in south-west Nigeria. Association between TDF-induced KTD and genetic polymorphisms in renal drug transporter genes and the *APOL1* (Apolipoprotein L1) gene will be examined.

**Methods:**

This study has two phases. An initial cross-sectional study will screen 3000 individuals attending the HIV clinics in south-west Nigeria for KTD to determine the prevalence and risk factors. This will be followed by a case-control study of 400 KTD cases and 400 matched controls to evaluate single nucleotide polymorphism (SNP) associations. Data on socio-demographics, risk factors for kidney dysfunction and HIV history will be collected by questionnaire. Blood and urine samples for measurements of severity of HIV disease (CD4 count, viral load) and renal function (creatinine, eGFR, phosphate, uric acid, glucose) will also be collected. Utility of urinary retinol binding protein (RBP) and N-acetyl-beta-D-glucosaminidase (NAG) levels as surrogate markers of KTD will be evaluated. Genomic DNA will be extracted from whole blood and SNP analyses performed using the rhAMP SNP genotyping assays. Statistical analysis including univariate and multivariate logistic regression analyses will be performed to identify factors associated with KTD.

**Discussion:**

In spite of TDF being the most commonly used antiretroviral agent and a key component of many HIV treatment regimens, it has potential detrimental effects on the kidneys. This study will establish the burden and risk factors for TDF-induced KTD in Nigerians, and explore associations between KTD and polymorphisms in renal transporter genes as well as *APOL1* risk variants. This study may potentially engender an approach for prevention as well as stemming the burden of CKD in sub-Saharan Africa where GDP per capita is low and budgetary allocation for health is inadequate.

## Background

Nigeria bears approximately 10% of the global burden of HIV/AIDS, with approximately 130,000 newly diagnosed HIV infections and 45,000 AIDS-related deaths reported in 2020, representing a 35% decrease from 2019 [1]. According to UNAIDS, 42% of the new HIV infections occur among couples practicing ‘low risk’ sex (heterosexual), while 40% occur among high-risk populations which constitute about 1% of the general population, including female sex workers, men that have sex with men and injecting drug users [2].

Both types of the human immunodeficiency virus, type 1 and 2, are present in Nigeria; a report of the analysis of 2228 western blot results showed that 98.3% were positive for HIV-1, 0.4% were positive for HIV-2 while 0.3% were co-infections [3].

According to the study by Oko-Jaja et al. [4], the prevalence of comorbid conditions among people living with HIV (PLWHIV) includes renal disease (14.4%), hypertension (6.2%), pulmonary tuberculosis (3.4%), hepatitis-B infection (1%), diabetes mellitus (0.6%), and hepatitis-C infection (0.2%). It is increasingly recognised that individuals of African descent are at a high risk of developing renal failure [5]. Chronic kidney disease (CKD) is at least 3–4 times more frequent in Africa than in developed countries [6] and HIV infection is associated with an increased risk of renal impairment, particularly in HIV individuals with pre-existing CKD [7, 8]. People living with HIV are not only at increased risk of HIV-related kidney disease, but are also prone to other causes of CKD, including diabetes and hypertension, observed in the general population [9].

According to 2020 data, 89% (comprising of 55% adults and 35% children) of those with a HIV positive diagnosis in Nigeria are accessing ART, and out of these, 42% were virally suppressed [1, 10]. Tenofovir disoproxil fumarate (TDF) is one of the most frequently used ART [11]. Currently, more than 80% of PLWHIV in Nigeria were receiving a TDF-based regimen, as Nigeria adopted TDF-based ART as the first-line treatment for HIV in 2014 [1, 12]. Whilst the initial randomised control trials suggest good overall safety for TDF, numerous subsequent studies have shown issues with the renal safety profile, including KTD in various study settings [13]. Furthermore, TDF has also been associated with a wide range of other manifestations of renal impairment than KTD such as low eGFR and rapidly declining eGFR. Globally, the reported incidence of TDF-induced nephrotoxicity ranges between 0.7 and 22% [14, 15]. Likanonsakul et al. [16] in their study reported that approximately 20% of PLWHIV receiving TDF in Thailand had β2-microglobulinuria, a marker of tubular dysfunction. Various risk factors have been associated with KTD including co-morbidities (such as hepatitis C, diabetes mellitus), low body weight, older age, concomitant administration of potentially nephrotoxic drugs, low CD4 count and duration of therapy [17].

In West Africa, a study carried out among a Ghanaian population of PLWHIV taking TDF showed that the prevalence of creatinine clearance less than 60 ml/min/1.73m^2^, dipstick proteinuria and asymptomatic renal tubular dysfunction was 7, 37 and 15% respectively [18]. In Nigeria, little is known about the prevalence of TDF-induced KTD. It is difficult to screen for this complication in resource-poor settings, and very few Nigerian studies have investigated markers of TDF-induced KTD. A study of 57 PLWHIV in Port Harcourt in southern Nigeria suggested an increased burden of KTD in individuals exposed to the TDF regimen. Results showed that after 12 weeks of exposure, 14% of PLWHIV exposed to TDF developed phosphaturia [19].

Renal tubular transporter proteins, including organic anion transporters (OAT) and multidrug resistance-associated proteins (MRP), otherwise known as the ATP binding cassette (ABC) superfamily, have been implicated in the uptake and secretion of TDF in the proximal renal tubule. Genetic variants in renal drug transporters are believed to confer susceptibility to TDF-induced KTD among Europeans [20], but an association between KTD and polymorphisms in genes encoding kidney drug transporters has not been previously investigated in African populations.

The mechanisms underlying the increased incidence of tubular injury in PLWHIV treated with TDF remain to be fully elucidated. Apolipoprotein L1 (APOL1), which accounts for almost 70% of the excess risk of kidney disease in individuals of African descent, is expressed in the proximal tubular cells in normal kidneys [21]. Approximately 87% of PLWHIV with CKD in Nigeria had 2 *APOL1* risk alleles, and previous studies have showed that *APOL1* genetic variants are strongly associated with HIV-associated nephropathy, a rapidly progressing kidney disease with severe tubular damage [22, 23]. Risk variants of *APOL1* act via a gain of injury or toxic activity on podocytes and other cells in the kidney including proximal tubular epithelial cells [21], thus *APOL1* renal risk alleles may contribute to the severity of TDF toxicity through this pathway.

In spite of TDF being the most commonly used antiretroviral agent and a key component of many HIV treatment regimens, it has been shown to have detrimental effects on the kidneys [13]. Tenofovir alafenamide (TAF), a novel TDF analogue with decreased renal injury, was recently approved by the US Food and Drug Administration [24], but the clinical significance of the long-term superior renal safety profile of TAF over TDF remains uncertain. As yet, there are no data for TAF safety in pregnancy, tuberculosis coinfection, or low CD4 count. It remains to be seen whether the substitution of TDF with TAF will make a justifiable incremental claim on scarce HIV treatment resources in the near future. There is increasing interest in the pharmacogenomics of renal drug transporters as a useful tool for identifying individuals who might be at risk of developing KTD, as it allows for a personalised approach to the use of TDF in PLWHIV on antiretroviral therapy.

This study aims to establish the burden and risk factors for TDF-induced KTD in Nigerians, and explore associations between KTD and polymorphisms in the renal transporter genes as well as *APOL1* risk variants. The study may potentially engender an approach for prevention as well as stemming the burden of CKD in sub-Saharan Africa particularly Nigeria, where GDP per capita is low and budgetary allocation for health is inadequate.

We hypothesise that KTD is common among adult PLWHIV in Nigeria who are receiving TDF-based antiretroviral therapy, and additive contributions from polymorphisms in kidney tubular transporters as well as *APOL1* genetic variants may be associated with the risk of KTD in Nigerians treated with TDF. Further, we hypothesise that the additive contribution from *APOL1* risk alleles would predispose to and enhance the severity of TDF toxicity and that the concomitant use of protease inhibitor may additively influence the susceptibility for severe TDF-induced nephrotoxicity. This paper describes the design and methodology for this study that aims to determine the prevalence and risk factors for TDF-induced KTD in PLWHIV in Nigeria.

## Methods/design

Primary outcomes of interest in this study are the prevalence of KTD and the association of polymorphisms in selected candidate SNPs in genes encoding tubular transporters and *APOL1* with KTD. Secondary outcomes include the presence of elevated retinol binding protein (RBP) levels (defined as retinol-binding protein/creatinine ratio > 2.93 μg/mmol) [25] and urinary N-acetyl-beta-D-glucosaminidase (NAG) levels (NAG > 5.93 U/g Cr).

### Study design

The prevalence and pharmacogenetics of TDF nephrotoxicity in adult PLWHIV in south-western Nigeria is a cross-sectional study with a case control sub-study of PLWHIV who are receiving TDF regimens attending anti-retroviral (ART) clinics in south-west Nigeria. This study will determine the prevalence of KTD in PLWHIV-I and II taking TDF in Nigeria. It will investigate the association of demographic (age, gender), lifestyle (BMI, smoking), concomitant medications (NSAIDs, protease inhibitors, traditional/herbal medicines) and concomitant illness (diarrhoeal disease, tuberculosis, pneumonia, hypertension, diabetes mellitus, HBV/HCV co-infection, etc.) with the development of TDF-induced KTD. This study will also explore whether polymorphisms in the *ABCB1, ABCC2, ABCC4, ABCC10, SLC22A6, SLC22A11* and *APOL1* genes are associated with KTD in PLWHIV in Nigeria treated with TDF, and explore the association between polymorphisms in genes encoding renal drug transporters and levels of urinary RBP as well as NAG in Nigerian PLWHIV treated with TDF. Recruitment into the study commenced in October 2019 and as of June 2020, 2491 participants have been enrolled.

### Setting and study population

The study will be conducted at the Department of Internal Medicine, Ladoke Akintola University of Technology Teaching Hospital (LTH), Osogbo, Nigeria and the Department of Internal Medicine Research Laboratory, University of the Witwatersrand, Johannesburg, South Africa. Participants will be recruited from ART clinics in Nigeria including LTH (Osogbo), Jaleyemi Catholic Hospital (Osogbo), Osun State Hospitals (Asubiaro, Ede, Ikire, Iwo, and Ila Orogun), Adeoyo State Hospital (Oyo State) and Seventh Day Adventist Hospital (Ile-Ife).

For the case-control phase of the study, PLWHIV on a TDF regimen under regular follow up for at least 3 months at the ART clinics who developed proximal KTD will be designated as cases. Research has shown that the median time to nephrotoxicity after TDF initiation is 3.6 months [8], hence, the rationale for a minimum of 3-months on TDF. The controls will be PLWHIV on a TDF regimen under regular follow up for ≥3 months at the ART clinics showing none of the abnormalities suggesting proximal KTD. The cases will be matched one-to-one to controls by gender, age (+/− 5 years), and TDF duration (+/− 3 months) and will be done on an ongoing basis during recruitment of study participants.

Participants meeting the inclusion criteria will be PLWHIV, aged 18 years and above, receiving TDF-containing therapy, on regular follow up for a minimum of 3 months with a viral load of less than 200 copies/ml. The rationale for the use of 3 months cut off point for the duration of tenofovir treatment was based on the results of a previous study by Brennan et al. [8] that showed that the median time to nephrotoxicity after initiation of TDF was approximately 3 months.

The following individuals will be excluded from the study: Individuals with incomplete history of antiretroviral treatment, including HIV treatment status (either treatment-naïve or experienced), date of commencement of ART and combination of ART); persons who are pregnant or lactating; persons with an estimated glomerular filtration rate (eGFR) < 60 ml/min/1.73m^2^ (eGFR will be calculated by Chronic Kidney Disease Epidemiology Collaboration [CKD-EPI] from serum creatinine estimated during the month enrolled in the study); persons with glomerulonephritis, including IgA Nephropathy and Lupus Nephritis; heart failure (NYHA 3 & 4), malignancy and non-Nigerians.

### Sample size and power analysis

At least 3000 participants will be recruited for the cross-sectional study from which 400 cases and 400 matched controls will be selected for the case-control sub-study. The sample size was calculated based on the following assumptions. Utilising 1000 Genomes data, we estimated the minor allele frequency (MAF) within the population, using the Yoruba in Ibadan (Nigeria) as a proxy for our study population [26]. The rare polymorphism, rs9349256 has the lowest MAF of 0.01 while the common polymorphism, rs717620 has the highest MAF of 0.42, the average MAF being 0.19. In addition, a Bonferroni correction was incorporated by setting our alpha level at 0.003 (0.05/18) to account for the multiple comparison testing. With the aid of a web browser program for basic sample size calculations of single nucleotide polymorphism (SNP) studies [27–29] [http://biostats.usc.edu/Quanto.html] and using a dominant inheritance model, the average MAF of 0.19, 13% KTD prevalence [30], a power of 80% and an alpha of 0.003, a sample size of 400 cases and 400 controls is able to detect a minimum odd ratio (OR) of 1.75 (case control phase). Table 1 indicates the required sample size (per group using a dominant model) to detect different odds ratios. Based on the data from a previous study in Nigerian PLWHIV which reported the prevalence of TDF-induced KTD as 13%, we made an assumption that 13% of TDF-treated individuals will develop KTD [30]. Therefore, taking this into consideration, the number needed to be screened to identify 400 cases is approximately 3000. We will therefore screen 3000 participants in the cross-sectional phase of this study.

**Table 1:** Calculations of the sample size (per group using a dominant model), required to detect different odds ratios in case-control analysis, assuming alpha = 0.003; power = 80% and ratio cases/controls = 1

Odds ratio	Sample Size (N)
1.25	2531
1.50	760
1.75	398
2.00	260
2.25	191
2.50	150
2.75	124
3.00	106

**Fig. 1:**
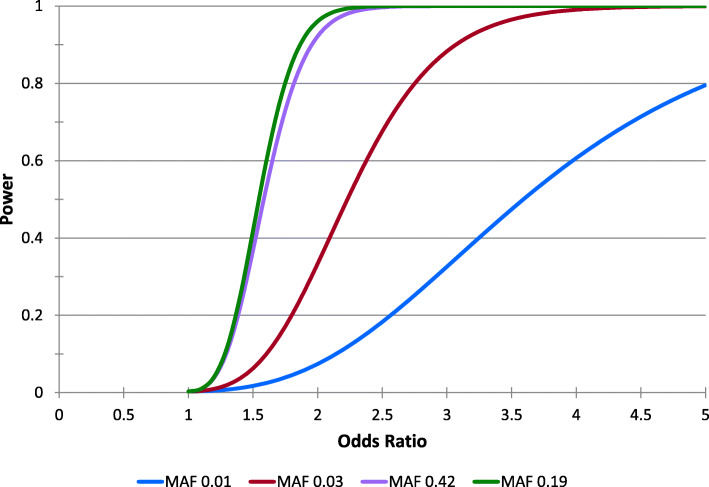
Model showing relationship between MAFs, odds ratio and power (*N* = 400)

Simulated trials to determine the power we have to detect our smallest and our largest MAFs, using a dominant inheritance model, and an alpha of 0.003, and a sample size of 400 showed that we are quite underpowered for the SNP with the lowest MAF; only able to reliably (power = 80%) find differences with an OR of 5 or greater. However, there is only one SNP with such a low MAF, and for the second lowest (MAF = 0.03) we have sufficient power to detect OR of 2.75 or greater. For the SNP with MAF = 0.42, we are powered to detect OR of just greater than 1.75 (Fig. 1).

### Recruitment of participants

**Fig. 2:**
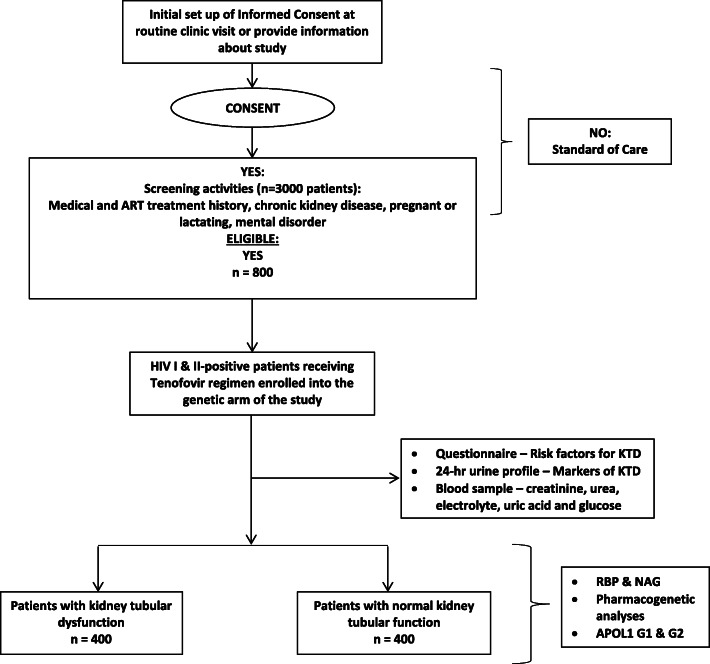
Study flow chart

An outline of how participants will be recruited for this study is shown in the study flow chart in Fig. 2. After obtaining patient consent, case report forms will be completed for all participants and will include the following data:

- Demographics - Age, sex, body weight, body mass index
- Past medical history - history of AIDS, route of HIV transmission, specific combination of ART, duration of therapy, history of tobacco use, hypertension, diabetes mellitus, congestive cardiac failure, co-infection with hepatitis B and C, concurrent use of ritonavir-boosted protease inhibitors, concurrent use of nephrotoxic drugs, such as ganciclovir and sulfamethoxazole/trimethoprim.
- Baseline laboratory data - CD4 count, HIV viral load, serum creatinine and estimated glomerular filtration rate (eGFR). As recommended by the National Kidney Foundation, eGFR will be calculated using the CKD-EPI formula [31].

### Diagnostic criteria for KTD

Kidney tubular dysfunction will be defined by the presence of at least 2 of the following abnormalities, with at least 1 being a Fanconi syndrome criterion (glucosuria in nondiabetic individuals or hyperphosphaturia) [32].

- Nondiabetic glucosuria (urine glucose level ≥ 300 mg/dL daily)
- Total excretion of phosphorus (urine phosphorus X urine volume) > 1200 mg daily
- Fractional tubular resorption of phosphorus (1- [(urine phosphorus X plasma creatinine)/(plasma phosphorus X urine creatinine)]) < 0.82
- Fractional excretion of uric acid ([(urine uric acid X plasma creatinine)/(urine creatinine X plasma uric acid)] X 100) > 15%
- Proteinuria > 0.2 g/24 h.

### Laboratory procedures

Blood and urine samples will be collected for routine laboratory measurements and the measurement of markers of renal function and proximal tubulopathy. The latter tests will be performed by LTH clinical laboratory services using an ADVIA® 1800 Chemistry System (Siemens, Germany). Blood samples will be used for creatinine, urea, electrolyte, uric acid and glucose measurements. Serum creatinine will be measured using the Jaffe method. A 24-h urine sample will be collected to measure protein, glucose, phosphate, uric acid and creatinine. Participants will be fasted overnight for at least 8 h and allowed to drink ad libitum. Spot urine samples will be collected and stored at − 80 °C for urinary RBP and NAG analysis using the RBP Urinary Competitive ELISA kit (ThermoFisher Scientific) and NAG ELISA Kit (R&D Systems, Inc. Minneapolis, USA).

An additional blood sample will be drawn for genomic DNA extraction. Samples will be collected into ethylenediaminetetraacetic acid (EDTA) vacutainers and stored at − 20 °C until DNA extraction. Genomic DNA will be extracted from the whole blood and the concentrations determined using a Qubit fluorometer (Thermo Fisher Scientific). DNA will be aliquoted and stored at − 80 °C.

The 18 SNPs to be genotyped are outlined in Table 2. Candidate SNPs were selected based on previously reported functional significance and minor allele frequencies > 5% in the literature [16, 32, 33]. SNPs will be genotyped using the rhAmp SNP Genotyping Assays. The rhAmp SNP assays provide a highly accurate, PCR-based genotyping method. The method is based on RNase H2-dependent PCR (rhPCR) and has improved specificity over TaqMan and KASP technologies [34]. The SNP genotyping will be carried out at the Internal Medicine Research Laboratory, University of Witwatersrand, South Africa.

**Table 2:** List of selected Single Nucleotide Polymorphisms

SNP	Gene	Chromosome	Position	Alleles
rs717620	ABCC2	Chromosome 10	99782821	C > T
rs2273697	ABCC2	Chromosome 10	99804058	G > A
rs17222723	ABCC2	Chromosome 10	99836239	T > A
rs3740066	ABCC2	Chromosome 10	99844450	C > T
rs8187710	ABCC2	Chromosome 10	99851537	G > A
rs899494	ABCC4	Chromosome 13	95209550	G > A
rs1751034	ABCC4	Chromosome 13	95062722	T > C
rs3742106	ABCC4	Chromosome 13	95021537	A > C
rs9349256	ABCC10	Chromosome 6	43404511	G > A
rs2125739	ABCC10	Chromosome 6	43445127	T > C
rs1045642	ABCB1	Chromosome 7	87509329	C > T
rs1128503	ABCB1	Chromosome 7	87550285	C > T
rs4149170	SLC22A6	Chromosome 11	62984817	C > T
rs11568626	SCL22A6	Chromosome 11	62984542	C > T
rs11231809	SCL22A11	Chromosome 11	64535478	T > A
rs73885319	APOL1	Chromosome 22	36265860	A > G
rs60910145	APOL1	Chromosome 22	36265988	T > G
rs71785313	APOL1	Chromosome 22	36265996-36266005	delTTATAA

### Statistical analysis

Descriptive results of continuous variables will be expressed as medians and interquartile ranges. Continuous variables will be tested for normality using the Shapiro–Wilk test and compared using the student’s *t* test or Wilcoxon rank sum test, as required. For the comparison of proportions, the Chi-square test will be used, with Fisher’s corrections applied when needed. Multivariate logistic regression analyses will be performed to identify factors associated with KTD. Parameters with significant *p*-values in the univariate analysis will be entered into a stepwise multivariate analysis. Deviations from Hardy-Weinberg equilibrium of individual alleles and differences in allele frequencies between the two groups will be evaluated by either the chi-square test or Fisher’s exact test where appropriate. Statistical comparisons for genotype frequencies between persons with KTD and those without KTD will be made by the chi-square test or Fisher’s exact test. Associations between allele sets or haplotypes and the risk of TDF-induced KTD will be estimated by computing odds ratios and 95% confidence intervals from multivariable logistic regressions. This analysis will include adjustments for demographic characteristics (age, sex, and BMI), baseline eGFR, duration of HIV infection, protease inhibitor therapy, and other antiretroviral treatment. All statistical analyses will be conducted using Stata 12 and R statistical software. *P* values < 0.05 will be considered statistically significant. Methods for controlling the false discovery rate and Bayesian analysis under shrinkage priors will be used when reporting the results of multivariable logistic regression.

## Discussion

Individuals of African descent have an increased risk of developing CKD, with CKD in non-HIV individuals 3–4 times more common in Africa than in developed countries [5, 6]. In Nigeria, 38–53% of PLWHIV develop CKD [35, 36]. The high prevalence of kidney disease in sub-Saharan Africa coupled with the initiation of TDF-based regimens in PLWHIV at risk of kidney failure highlights the need to regularly screen PLWHIV for renal dysfunction, including KTD to reduce the burden of nephrotoxicity.

There has been increasing interest in the pharmacogenomics of renal drug transporters as a useful tool for identifying individuals who might be at risk of developing KTD, as it allows for a personalised approach to the use of TDF in PLWHIV on antiretroviral therapy. However, it is difficult to screen for genetic markers of KTD in resource poor areas, and to date no African study has investigated genetic markers of KTD. Moreover, previous studies on the pharmacogenetics of KTD in PLWHIV who were treated with TDF have provided diverse results [32, 33, 37]. Several factors could induce KTD, including infection, inflammation, pre-existing kidney disease, concurrent use of nephrotoxic drugs and diabetic kidney disease, and it is therefore difficult to evaluate whether KTD is exclusively caused by TDF. Thus, the role of genetic polymorphisms in drug transporter genes on TDF-induced KTD remains to be distinguished and isolated from other aforementioned risk factors for KTD, especially in individuals of African descents.

Based on this background, this study was designed to identify those risk factors that may be associated with TDF-induced KTD in Nigerian PLWHIV who are receiving a TDF regimen with suppressed viral load, and free of pre-existing kidney impairment and major comorbidities. This study also seeks to investigate the interaction between these risk factors and genetic predisposition as well as the interaction between polymorphisms in drug transporter genes and *APOL1* genetic variants.

Owing to the lack of adequate treatment of CKD in resource-limited settings, it is critical to prevent or slow progression to end-stage kidney disease. The public-health implications of early detection of renal dysfunction in PLWHIV are huge, thus allowing for development of a reliable strategy to detect and preferably avoid tenofovir-associated kidney toxicity. The lessons learnt from this study can be utilized for the development of clinical management programs to reduce the burden of CKD in Nigeria, and particularly in sub-Saharan Africa where GDP per capita is low and budgetary allocation for health is inadequate.

### Strengths and limitations

This study will be the first to evaluate the role of renal transporters gene polymorphisms on TDF-induced KTD in an indigenous black African population. The possible additive contribution from *APOL1* gene polymorphisms to the severity of KTD will be also be evaluated. A limitation of the study is that there will be no longitudinal data collection to assess KTD progression. However, several additional in-depth analyses will be undertaken on this cohort, building on the observations in this paper. Among the planned work is an in-depth analysis of follow-up time on TDF and longitudinal analyses of association of KTD with eGFR and worsening proteinuria. Even though we made extensive efforts to exclude potential confounding variables in this study, the effect of unmeasured confounders is still possible. In addition, the absence of whole genome sequencing technologies means that variations in the African genome will not be captured.

## Supplementary information

**Additional file 1:.**

## Data Availability

Not applicable.

## Abbreviations

AA: African American
ABC: ATP binding cassette
AIDS: Acquired immune deficiency syndrome
APIN: AIDS prevention initiative in Nigeria
*APOL1*: Apolipoprotein L1
ART: Anti-retroviral therapy
BMI: Body mass index
CD4: Cluster of differentiation
CKD: Chronic kidney disease
CKD-EPI: Chronic Kidney Disease Epidemiology Collaboration
DNA: Deoxyribose nucleic acid
eGFR: Estimated glomerular filtration rate
GDP: Gross domestic product
HIV: Human immunodeficiency virus
KTD: Kidney tubular dysfunction
LTH: Ladoke Akintola University Technology Teaching Hospital
MAF: Minor allele frequency
MRP: Multidrug resistance-associated proteins
NAG: N-acetyl-beta-D-glucosaminidase
NYHA: New York Heart Association
NSAIDs: Non-steroidal anti-inflammatory drugs
OAT: Organic anion transporters
PCR: Polymerase chain reaction
PLWHIV: People living with HIV
SNP: Single nucleotide polymorphisms
TAF: Tenofovir alafenamide
TDF: Tenofovir disoproxil fumarate
