# Supplementary material for: Design and methods of the prevalence and pharmacogenomics of tenofovir nephrotoxicity in HIV-positive adults in south-western Nigeria study
Source: BMC Nephrol. 2020 Oct 16;21:436. doi: 10.1186/s12882-020-02082-3 (PMC7565751; doi:10.1186/s12882-020-02082-3)
Supplement: Supplementary file 1 — Additional file 1:. [file 12882_2020_2082_MOESM1_ESM.doc]

**CASE REPORT FORM (CRF)**

**TITLE:**  PREVALENCE AND PHARMACOGENOMICS OF TENOFOVIR NEPHROTOXICITY IN HIV-POSITIVE ADULTS IN SOUTH-WESTERN NIGERIA

**STUDY NUMBER:** LTH/EC/2017/05/316

**PRINCIPAL INVESTIGATOR:** DR MUZAMIL HASSAN

1. GENERAL INFORMATION

| IDENTIFICATION NUMBER |  |
| --- | --- |
| ENROLLMENT DATE |  |

1. DEMOGRAPHICS AND ANTHROPOMETRIC PARAMETERS

| AGE (YEARS) |  |
| --- | --- |
| SEX | MALE  FEMALE |
| WEIGHT(KG) |  |
| HEIGHT (CM) |  |
| BODY MASS INDEX (KG/M^2^) |  |

1. MEDICAL HISTORY

| SMOKING | SMOKER  FORMER SMOKER  NON-SMOKER |
| --- | --- |
| HEPATITIS B INFECTION | YES  NO |
| HEPATITIS C INFECTION | YES  NO |
| HYPERTENSION | BLOOD PRESSURE: |
| DIABETES MELLITUS | YES  NO |
| HEART FAILURE | YES  NO |
| CONCOMITANT MEDICATIONS | SULFAMETHOXAZOLE/TRIMETHOPRIM  NSAIDs  ACYCLOVIR  CIDOFOVIR  ADEFOVIR  GANCICLOVIR |
| TRADITIONAL/HERBAL MEDICINES | YES  NO |
| CONCOMITANT ILLNESS | DIARRHOEAL DISEASE  PULMONARY TUBERCULOSIS  PNEUMONIA |
| DATE OF DIAGNOSIS AND TIME SINCE DIAGNOSIS OF HIV ILLNESS |  |
| ROUTE OF HIV TRANSMISSION |  |
| HAART START DATE |  |
| ANTIRETROVIRAL REGIMEN | EMTRICITABINE  LAMIVUDINE  ZIDOVUDINE  DIDANOSINE  ABACAVIR  LOPINAVIR  RITONAVIR  EFAVIRENZ  NEVIRAPINE  TENOFOVIR  RITONAVIR-BOOSTED PIs |
| TENOFOVIR START DATE |  |
| EVER RECEIVED A PROTEASE INHIBITOR BEFORE TENOFOVIR? | YES  NO |
| BASELINE CD4 COUNT |  |

1. BLOOD TESTS

| TESTS |  |
| --- | --- |
| HAEMOGLOBIN |  |
| GLUCOSE |  |
| CREATININE |  |
| UREA |  |
| URIC ACID |  |
| PHOSPHORUS |  |
| ESTIMATED GFR |  |

1. URINE TESTS

| PROTEIN |  |
| --- | --- |
| GLUCOSE |  |
| PHOSPHATE |  |
| URIC ACID |  |
| RETINOL BINDING PROTEIN |  |
| N-ACETYL-B-D-GLUCOSAMINIDASE |  |

1. GENE POLYMORPHISM

| ABCC2 −24C→T (rs717620) |  |
| --- | --- |
| ABCC2 −1249 G→A (rs2273697) |  |
| ABCC2− 3563 T→A (rs8187694) |  |
| ABCC2 −3972 C→T (rs3740066) |  |
| ABCC2 −4544 G→A (rs8187710) |  |
| ABCC4 −669 C→T (rs899494) |  |
| ABCC4 −3463 A→G (rs1751034) |  |
| ABCC4 −4131 T→G (rs3742106) |  |
| ABCC10 −526 G → A (rs9349256) |  |
| ABCC10 −2843 T → C (rs2125739) |  |
| ABCB1 −3435 C→T (rs1045642) |  |
| ABCB1 −1236 C→T (rs1128503) |  |
| SLC22A6−453 G → A (rs4149170) |  |
| SLC22A6−728 G → A (rs11568626) |  |
| SLC22A11− rs11231809 |  |
| APOL1 (rs73885319) |  |
| APOL1 (rs60910145) |  |
| APOL1 (rs71785313) |  |
